# Editorial: The complex phenotype of diabetic cardiomyopathy: clinical indicators and novel treatment targets

**DOI:** 10.3389/fendo.2024.1497352

**Published:** 2024-09-30

**Authors:** Priyanka Choudhury, Ramoji Kosuru, Yin Cai

**Affiliations:** ^1^ Department of Cell Biology, Neurobiology and Anatomy, Medical College of Wisconsin, Milwaukee, WI, United States; ^2^ Vascular Signaling, Versiti Blood Research Institute, Milwaukee, WI, United States; ^3^ Department of Health Technology and Informatics, The Hong Kong Polytechnic University, Hong Kong, Hong Kong SAR, China

**Keywords:** diabetic cardiomyopathy, cell death, inflammation, oxidative stress, signaling pathway

This Research Topic highlights the complex phenotype of diabetic cardiomyopathy (DCM), focusing on its clinical indicators and potential treatment approaches. DCM is a severe complication of both type 1 and type 2 diabetes, characterized by myocardial fibrosis, impaired cardiac function, and increased mortality rates among diabetic patients. The estimated global prevalence of diabetes is expected to reach 12.2% (783.2 million people) by 2045, with a significant percentage of diabetic individuals (11.7% to 67%) developing DCM based on varying diagnostic criteria (1, 2). Moreover, diabetic patients have a 2.45 to 2.99 times higher incidence of myocardial ischemia compared to non-diabetics (3). Therefore, accurate estimates of current and future prevalence of type 2 diabetes are crucial for effective health care planning and targeted interventions to reduce risk factors and reverse increasing trends. However, projections such as those from the International Diabetes Federation’s Diabetes Atlas often emphasize urbanization and demographic shifts, overlooking other crucial risk factors like obesity and smoking. To address this, Moreira et al. used the IMPACT TYPE 2 DIABETES model, which incorporates demographic changes along with obesity and smoking trends using a Markov approach in the Brazilian population. Their findings predict a rise in diabetes prevalence, even with aggressive obesity reduction strategies, highlighting the crucial role of tackling obesity to prevent diabetes. The study calls for expanding current initiatives to make a significant impact, suggesting that with stronger efforts, it is possible to lower type 2 diabetes prevalence in line with national and international policies.

Arterial stiffness (AS) is a key factor in the development of cardiovascular disease (CVD), serving as an early indicator of atherosclerosis and a predictor of CVD risk and mortality (4). Lifestyle choices, including sedentary habits and diets rich in processed carbohydrates and saturated fats, contribute to conditions like obesity, lipid disorders, and insulin resistance, which in turn exacerbate AS. Early detection of AS is particularly important for individuals type 2 diabetes, as they face a higher risk of cardiovascular complications. In response to this, Mao et al. investigated the relationship between lipid accumulation product (LAP) index—based on waist circumference and triglyceride levels—and brachial-ankle pulse wave velocity (baPWV), a key AS indicator, in Chinese type 2 diabetic patients. The study revealed a strong positive correlation between LAP and baPWV, which persisted even after adjusting for various factors and was consistent across different genders and subgroups. This suggests that LAP could be useful tool for assessing AS risk in clinical settings and research.

Additionally, Zeng et al. explored the causal relationship between 1,400 metabolites and dilated cardiomyopathy through a two-sample Mendelian randomization (MR) approach. They identified 52 metabolites with causal association to the disease, some of which were positively linked, while others were negatively correlated. Elevated levels of tryptophan betaine and 5-methyluridine were found to increase the risk of dilated cardiomyopathy, whereas myristoleate and erythronate were linked to a reduced risk. These insights into metabolic factors offer promising avenues for new treatment approaches and biomarker development to improve disease management.

Understanding the underlying causes and pathology of DCM is essential for the development of new drug therapies and clinical indicators. Key contributors to DCM progression include oxidative stress, inflammation, and cell death, with cardiomyocyte death playing a central role. Various forms of cell death – such as apoptosis, autophagy, necrosis, ferroptosis, and pyroptosis – are implicated in the disease. Ferroptosis, a type of programmed cell death driven by iron accumulation and lipid peroxidation, results from deficiencies in oxidoreductases such as glutathione peroxidase 4, which diminishes cellular antioxidant defenses and contributes to myocardial dysfunction (3). Similarly, pyroptosis, a recently identified inflammatory form of programmed cell death, is crucial in DCM progression. This Research Topic includes two key reviews exploring the roles of ferroptosis and pyroptosis in DCM. Zhao et al. discuss the molecular mechanisms linking ferroptosis to DCM and evaluate potential therapeutic approaches using ferroptosis inducers and inhibitors. Wang et al. provide an overview of the mechanisms by which pyroptosis contributes to DCM and explore targeted treatments that focus on NLRP3 inflammasome pathway. Both reviews highlight the importance of understanding these processes, which may lead to new drug developments that can slow or reverse DCM progression, ultimately improving patient outcomes.

Current management of DCM focuses on controlling blood glucose and lipid levels, but no specific or reliable drugs are available for the condition. Other CVD risk factors, such as myocardial infarction (MI), dyslipidemia, and hypertension, also play critical roles in DCM progression. Among these, acute myocardial infarction (AMI) is a leading cause of cardiovascular death worldwide (5). As a result, identifying effective treatments for MI and improving its prognosis are essential for reducing cardiovascular mortality and enhance global health outcomes. In this context, Zhuang et al. conducted a Mendelian randomization analysis to explore the genetic relationship between the metformin use and various MI outcomes. Their findings revealed that metformin does not reduce the risk of acute transmural MI of the anterior wall and might increase the risk of overall MI, old MI, acute MI, and acute transmural MI of the inferior wall. This suggest that metformin may not provide the expected protection against MI and could even increase the risk for various forms of MI.

A meta-analysis by Wang et al. on dipeptidyl peptidase-4 inhibitors (DPP4i) revealed their potential benefits in improving cardiac structure and function, highlighting DPP4i as a promising treatment for preventing MI and other CVDs. Additionally, using a network pharmacology approach, another study by Wang et al. identified the SIRT1, Nrf2, and NQO1 signaling pathways as targets for YuNü-Jian, a traditional Chinese medicinal formula, suggesting its potential for managing DCM. Moreover, Jiang et al. reviewed the cardioprotective properties of salvianolic acid in diabetic patients, particularly its ability to protect against myocardial ischemia-reperfusion injury. The beneficial effects of salvianolic acid are linked to its modulation of oxidative stress, inflammation, mitochondrial dysfunction, ferroptosis, and apoptosis through key pathways such as PI3K/Akt, JAK/STAT, and NF-kB. These findings point to salvianolic acid’s promising potential in cardiovascular protection for diabetic patients.

The current research findings offer promising insights, but further detailed clinical studies are critical to effectively address DCM and other CVDs. There is also an urgent need for reliable and sensitive clinical markers to detect early cell death in DCM. Currently, the absence of clear diagnostic criteria for DCM poses challenges in differentiating myocardial injury, hemodynamic changes, and reduced cardiac function caused by cell death from those resulting from other conditions, such as coronary atherosclerosis or ischemic cardiomyopathy. In evaluating the efficacy of treatment for DCM, it is important to consider factors such as drug dosage, timing of administration, and patient characteristics including age, gender, and the presence of comorbidities. Therefore, developing strategies to mitigate risk and intervene in DCM progression is of great clinical and societal importance, especially as it may improve outcomes for those at risk of cardiovascular complications.
